# Holmium laser enucleation of a 696 cc prostate (HoLEP): The largest reported in the literature^☆^

**DOI:** 10.1016/j.eucr.2022.102203

**Published:** 2022-08-24

**Authors:** Shahed Borojeni, Fares Kosseifi, Axel Dallongeville, Xavier Durand

**Affiliations:** a Department of Urology, Groupe Hospitalier Paris Saint-Joseph, Paris, France; b Department of Radiology, Groupe Hospitalier Paris Saint-Joseph, Paris, France

**Keywords:** HoLEP, BPH, Male LUTS, Prostate, Endourology, World record, BPH, benign prostatic hyperplasia (BPH), GPH, giant prostatic hyperplasia, HoLEP, Holmium Laser Enucleation of the Prostate (HoLEP), IPSS, International Prostate Symptom Score, LUTS, Lower Urinary Tract Symptoms, MRI, Magnetic resonance imaging

## Abstract

Laser enucleation of the prostate represents the endoscopic response to open simple prostatectomy for the treatment of large benign prostatic hyperplasia (BPH) and an advanced technique for prostate surgery. To date, no more than 20 cases of giant prostatic hyperplasia (GPH) have been reported in the literature. We report a successful holmium laser enucleation of a 696 cc prostate in a 78 year-old patient on anticoagulation that was embolized prior to the intervention, urinary catheter was removed on the first day post-operatively and the patient was discharged home on the second day. This is considered the largest prostate treated endoscopically.

## Highlights

• HoLEP is feasible for GPH and is thus volume independent.
• HoLEP is a safe and a less morbid alternative for open simple prostatectomy.
• Multidisciplinary discussion and approaches are needed even for non-oncological cases.
• Six hundred and ninety-sixth grams is now the world Record for HoLEP.

## 1 Introduction

Laser enucleation of the prostate represents the endoscopic response to open simple prostatectomy for the treatment of large benign prostatic hyperplasia (BPH) and an advanced technique for prostate surgery. BPH is considered the most frequent but not the only cause of lower urinary tract symptoms (LUTS). Giant prostatic hyperplasia (GPH) is defined as BPH over 500 cc.^1^ To date, no more than 20 cases of GPH are reported in the literature, and the largest to be treated by holmium laser enucleation (HoLEP) was 400 cc.^2^ We report a HoLEP of a 696 cc GPH. To our knowledge this is the largest prostate ever to be treated endoscopically.

## 2 Case presentation

A 78 year-old patient presented for a BPH with long lasting moderate storage and voiding urinary symptoms (IPSS >7 and a poor quality of life) refractory to dual medical therapy (silodosin and finasteride) and recently complicated by recurrent bacterial prostatitis and transient acute urinary retention. The digital rectal exam revealed a giant prostate with a smooth palpable apex.

It's worth noting that he has a stented coronary artery disease and is on oral anticoagulation with Rivaroxaban for an atrial fibrillation.

His urinalysis was sterile. PSA level was elevated (41 ng/mL) with otherwise unremarkable laboratory values. PSA density was 0.059 ng/ml^2^ (<0.15 mg/ml/cc) in favor of BPH.

A multiparametric MRI of the prostate was performed by our uro-radiologist and showed a GPH of 696 cc, without suspicious lesions. Important neoangiogenesis with multiple prostatic microaneurysms were noted as well as a mild left uretero-hydronephrosis seen on an abdomen and pelvis CT angiogram. No suspicious lesions or lymphadenopathies were seen.

Flexible cystoscopy showed a massively obstructive prostate without any suspicious bladder lesion (the distance between urethral meatus and the bladder neck was 32 cm).

After a multidisciplinary team discussion, a decision to omit prostatic biopsies was taken for many reasons:

1 Low PSA density alongside a normal 3T mpMRI of the prostate (no PIRADS ≥3 lesions).
2 Age of the patient, anticoagulation, comorbidities and life expectancy.
3 The chief complaint was altered quality of life due to male LUTS.
4 Low sampling capacity of standard 10–12 core biopsies in such a large prostate.
5 Even if localized prostate cancer was diagnosed (no metastatic lesions on MRI and CT), and according to guidelines, a radical surgical treatment is not the first choice for this patient but rather radiotherapy ( ± ADT), if any (watchful waiting is an option). In addition, radiotherapy cannot be performed for this patient before prior treatment of his BPH.

A decision to perform a HoLEP was made with perioperative bridging with low-molecular-weight heparin, alongside a pre-surgical prostatic embolization the day before the surgery.

The aim of pre-surgical embolization was to minimize intraoperative bleeding which even if instantly minimal, could be cumulated and significant because of the predicted duration of the surgery especially in a cardiac comorbid patient. In addition, this strategy could improve endoscopic vision and optimize surgical conditions.

**Fig. 1:**
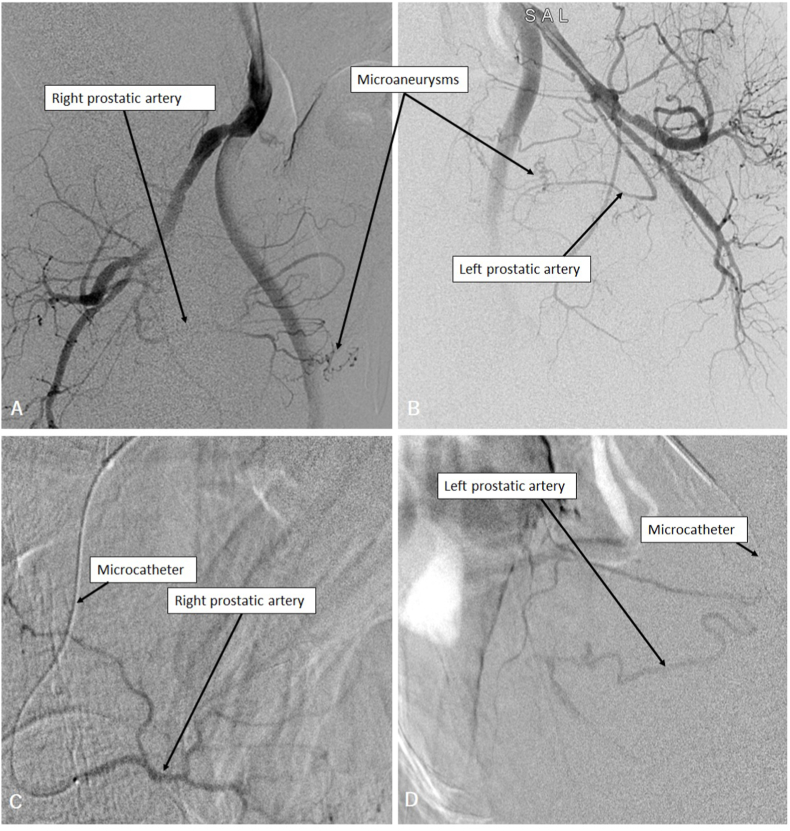
Right (A) and left (B) internal iliac arteries angiography showing enlarged prostatic arteries and vascular microaneurysms. Microcatheterization of the right (C) and left (D) prostatic arteries prior to embolization.

Arterial embolization of the two main prostatic arteries was successfully performed through a left radial access (Fig. 1).

HoLEP was performed using a 550-μm holmium-YAG laser fiber and the three-lobe enucleation technique. An energy of 75 W (1.5 J - 50 Hz) was used for enucleation and 40 W (2 J - 20 Hz) for hemostasis. The total operative time was 4 hours 50 minutes for a totally delivered energy of 540 KJ (381 KJ for enucleation, 159 KJ for hemostasis). Enucleation time was 2 hours 40 minutes, morcellation was performed after enucleation of each lobe separately due to the limited bladder space and took 65 minutes in total and hemostasis was performed in 50 minutes (Video 1). A total of 64 irrigation bags (3 L each) of normal saline were consumed. The intraoperative bleeding was moderate, however requiring an intraoperative transfusion of one unit of packed red blood cell. Bilateral externalized 7Fr ureteral catheters were inserted at the end of the procedure, due to the proximity of ureteral orifices to the huge enucleation fossa, alongside a 22Fr three-way urinary catheter with clear normal saline irrigation at the end of the procedure.

Supplementary video related to this article can be found at https://doi.org/10.1016/j.eucr.2022.102203

The following is the supplementary data related to this article:

Video Video-endoscopic recording of the procedure. Video

Both ureteral catheters as well as the urinary catheter were removed on day 1 post-operatively. Postoperative hemoglobin was 11 g/dl and the GFR was 89 ml/min. The patient voided successfully (residual volume <50 ml), low-molecular-weight heparin was resumed on day 1 and Rivaroxaban at 3 weeks. The patient was discharged home on day 2 post operatively after an additional 24 hours surveillance.

Histopathologic results showed nodular hyperplasia without malignant changes.

**Fig. 2:**
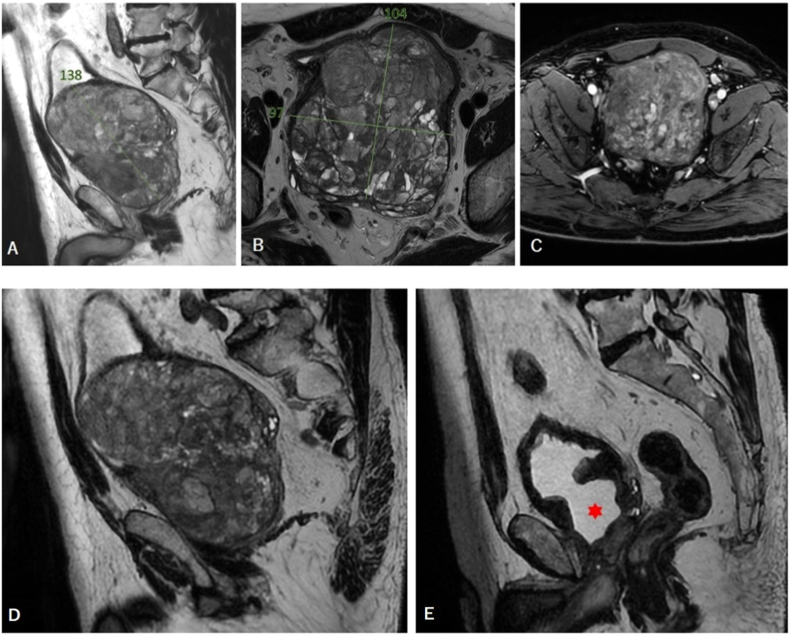
Sagittal T2 (A), axial T2 (B) and axial T1 with fat suppression (C) cuts on a pre-operative multiparametric MRI of the prostate. A pre- (D) and post-operative (E) prostate MRI-based comparison on a sagittal view showing the empty prostatic fossa (red star) at 2 months after HoLEP. (For interpretation of the references to colour in this figure legend, the reader is referred to the Web version of this article.)

Two months post-operative follow-up showed a significant clinical improvement of the urinary stream on uroflowmetry and both storage and voiding urinary symptoms (IPSS <7). However, the patient complained of a mild intermittent stress urinary incontinence for which pelvic floor exercises were prescribed. No post void residual volume was noted. A comparative MRI showed an empty prostatic fossa with a 13 cc residual prostatic tissue (Fig. 2). Post-operative PSA was 0.2 ng/ml.

## 3 Discussion

HoLEP was developed in the 1990s. Since then, it has undergone significant changes: advancements in laser technology, endoscopic morcellation devices, and surgical techniques.^3^

Today's technological improvements make it an effective, safe and less morbid endoscopic treatment for BPH, even in anticoagulated patients.

Surgeons HoLEP experience has improved despite the persistent steep learning curve. These advances made this technique volume independent and pushed further the boundaries of endoscopic treatment for BPH.^4^

According to the latest European Association of Urology guidelines on non-neurogenic male LUTS, HoLEP can be offered as an alternative for transurethral resection (previously considered the Gold Standard for volumes <80 ml) and for open prostatectomy (previously considered the Gold Standard for volumes ≥80 ml).^5^

## 4 Conclusion

Our center experience with HoLEP (>300/year) allowed us to perform this intervention in an extreme case. The immediate pre-surgical embolization was tested as a sandwich treatment in an attempt to decrease operative bleeding. Perioperative results and follow-up were satisfying encouraging us to publish this case and share our experience.

## Funding

This research did not receive any specific grant from funding agencies in the public, commercial, or not-for-profit sectors.
